# Supplementary material for: Estimating the health impacts of sugar-sweetened beverage tax for informing policy decisions about the obesity burden in Vietnam
Source: PLoS One. 2023 Apr 4;18(4):e0274928. doi: 10.1371/journal.pone.0274928 (PMC10072454; doi:10.1371/journal.pone.0274928)
Supplement: S1 File — (DOCX) [file pone.0274928.s001.docx]

# SUPPLEMENTARY 1: Data inputs and assumptions

**Table S1** describes the main model inputs and assumptions with specific clarifications. Data for these inputs and assumption were drawn from the desk review and various available data sources as indicated.

**Table S1: Model parameters & data sources**

| Indicators | Description | Data sources |  |
| --- | --- | --- | --- |
| Step 1: Change in SSB consumption | | | |
| Price elasticity | A parameter for estimating the change of SSB purchasing and consuming of when the price is increased from the taxation. | Linh Luong et al 2020 (1); literature review |  |
| Tax increase (%) | The relative change of SSB tax rate compared to the baseline rate to set up different tax scenarios | Shared data from the joint work of WHO & Health Bridge Canada |  |
| Baseline SSB consumption (liter) | Amount of SSB consumption in year 2020 separated by 4 categories (carbonates, juices, ready-to-drink tea/ coffee & sport drinks) | Global Data 2021 |  |
| SSB consumption per capita (100ml/ day/ person) | Amount of SSB consumption per capita in year 2020, separated by 4 categories (carbonates, juices, ready-to-drink tea/ coffee, energy & sport drinks) | Author’s calculation for population age 2+ (using GSO Census 2019) |  |
| Population size | Population structure by age and sex, also representing the market size of SSB in Vietnam | GSO Census 2019 |  |
| Step 2: Change in energy intake | | | |
| Sugar content | The average amount of sugar contained in each SSB lines, measured in gram/ 100ml | Paraje G. (WHO’s expert report) (2) |  |
| Calorie density | The average calorie density in each SSB categories, measured in kJ/100ml | Author’s calculation from sugar content |  |
| Baseline energy intake | The average daily intake of calories at baseline, measured as kJ/day/person | National Nutrition Survey 2019-2020 |  |
| Step 3: Change in body weight, BMI & obesity status | | | |
| Conversion factors for calorie-to-weight | The corresponding change of weight due to the decreased intake of calorie consumed for children and adults | Literature review  (see more in Method section above) |  |
| Baseline weight | The baseline weight of population by sex & age | STEP 2015 |  |
| Baseline height | The baseline height of population by sex & age | STEP 2015 |  |
| Baseline BMI | The baseline BMI of population by sex & age | STEP 2015 |  |
| BMI change | The absolute change between baseline BMI and the counterfactual BMI | Cohort simulation using STEP 2015 cohort |  |
| Step 4: Change in burden of obesity-related diseases | | | |
| Risk reduction for diabetes | The relative reduction in risk of diabetes mellitus type 2 for each 2 kg/m2 BMI unit decrease | Asia Pacific Cohort Studies Collaboration 2005 (3) |  |
| % of diabetes with complications | % of diabetes patients with complications | VSS 2017 (Kiet Pham et al 2020 (4)) |  |
| Medical cost for diabetes with complications | The average treatment cost of diabetes patients with complications, inflation adjusted to 2020 price (USD) | VSS 2017 (Kiet Pham et al 2020 (4)) |  |
| Medical cost for diabetes without complications | The average treatment cost of diabetes patients with complications, inflation adjusted to 2020 price (USD) | VSS 2017 (Kiet Pham et al 2020 (4)) |  |

**References**

1. Luong L, Vu LH. Impacts of Excise Taxation on Non-Alcoholic Beverage Consumption in Vietnam. Sustainability. 2020;12(3):1092.

2. Guillermo Paraje. Fiscal tools for reducing the consumption of sugar-sweetened beverages in Vietnam (Consultancy report). Hanoi: World Health Organization; 2016.

3. Ni Mhurchu C, Parag V, Nakamura M, Patel A, Rodgers A, Lam TH. Body mass index and risk of diabetes mellitus in the Asia-Pacific region. Asia Pac J Clin Nutr. 2006;15(2):127-33.

4. Tuan Kiet Pham H, Tuyet Mai Kieu T, Duc Duong T, Dieu Van Nguyen K, Tran NQ, Hung Tran T, et al. Direct medical costs of diabetes and its complications in Vietnam: A national health insurance database study. Diabetes Res Clin Pract. 2020;162:108051.
