# Supplementary material for: Estimating the health impacts of sugar-sweetened beverage tax for informing policy decisions about the obesity burden in Vietnam
Source: PLoS One. 2023 Apr 4;18(4):e0274928. doi: 10.1371/journal.pone.0274928 (PMC10072454; doi:10.1371/journal.pone.0274928)
Supplement: S2 File — (DOCX) [file pone.0274928.s002.docx]

# **SUPPLEMENTARY 2: Sensitivity analysis result**

**Table S2: Deterministic sensitivity analysis on price elasticity at -1.0**

| Tax design | | Scenario 1 | Scenario 2 | Scenario 3 | Scenario 4 | Scenario 5 | |
| --- | --- | --- | --- | --- | --- | --- | --- |
|  |  | *10% ad-valorem* | *3,500d/ liter* | *60d/ gr/100ml* | *7,000d/ liter* | *40% ad-valorem* | |
| Weight change (kg) | | | | | | |  |
| *Children aged 2-17* | Boys | 0.07 | 0.14 | 0.28 | 0.27 | 0.29 | |
|  | Girls | 0.08 | 0.15 | 0.30 | 0.29 | 0.31 | |
| *Adults aged 18+* | Male | 0.13 (0.01) | 0.25 (0.01) | 0.51 (0.03) | 0.50 (0.03) | 0.52 (0.03) | |
|  | Female | 0.17 (0.01) | 0.32 (0.02) | 0.66 (0.03) | 0.64 (0.03) | 0.67 (0.03) | |
| Projected overweight prevalence (23 ≤ BMI <25) – Mean (SE) | | | | | | |  |
| Both sexes | | 17.7% (0.88%) | 17.0% (0.85%) | 16.5% (0.83%) | 16.5% (0.83%) | 16.4% (0.82%) | |
| Male | | 19.5% (1.37%) | 18.5% (1.33%) | 18.0% (1.32%) | 18.1% (1.32%) | 17.8% (1.32%) | |
| Female | | 15.9% (0.98%) | 15.6% (0.99%) | 15.0% (0.95%) | 15.0% (0.95%) | 14.9% (0.95%) | |
| Projected obesity grade I prevalence (25 ≤ BMI <30) – Mean (SE) | | | | | | |  |
| Both sexes | | 13.2% (0.81%) | 13.0% (0.82%) | 12.7% (0.82%) | 12.7% (0.82%) | 12.7% (0.82%) | |
| Male | | 12.6% (1.19%) | 12.5% (1.19%) | 12.4% (1.18%) | 12.4% (1.18%) | 12.4% (1.18%) | |
| Female | | 13.8% (1.02%) | 13.4% (1.01%) | 13.1% (1.01%) | 13.1% (1.01%) | 13.1% (1.01%) | |
| Projected obesity grade II prevalence (BMI ≥30) – Mean (SE) | | | | | | |  |
| Both sexes | | 3.4% (0.44%) | 3.3% (0.43%) | 3.3% (0.43%) | 3.3% (0.43%) | 3.3% (0.43%) | |
| Male | | 1.6% (0.37%) | 1.6% (0.37%) | 1.6% (0.37%) | 1.6% (0.37%) | 1.6% (0.37%) | |
| Female | | 5.1% (0.76%) | 5.0% (0.74%) | 4.9% (0.74%) | 4.9% (0.74%) | 4.9% (0.74%) | |
| % reduction in diabetes prevalence | | | | | | |  |
| Both sexes | | 0.03% (0.000%) | 0.05% (0.000%) | 0.09% (0.001%) | 0.08% (0.001%) | 0.09% (0.001%) | |
| Male | | 0.01% (0.000%) | 0.02% (0.000%) | 0.04% (0.001%) | 0.04% (0.001%) | 0.04% (0.001%) | |
| Female | | 0.04% (0.000%) | 0.07% (0.001%) | 0.12% (0.001%) | 0.12% (0.001%) | 0.12% (0.001%) | |
| Number of avoided diabetes case | | 17,804  (88) | 34,023 (176) | 56,080 (264) | 54,437 (264) | 56,989 (264) | |
| Healthcare cost saving (mil. USD) | | 6.11 | 11.68 | 19.25 | 18.69 | 19.56 | |
|  |  | (0.04) | (0.07) | (0.12) | (0.12) | (0.12) | |
| Healthcare cost saving (bil. VND) | | 136.71 | 261.26 | 430.64 | 418.02 | 437.62 | |
|  |  | (0.89) | (1.57) | (2.68) | (2.68) | (2.68) | |

**Table S3: Deterministic sensitivity analysis on price elasticity at -0.8**

| Tax design | | Scenario 1 | Scenario 2 | Scenario 3 | Scenario 4 | Scenario 5 |
| --- | --- | --- | --- | --- | --- | --- |
|  |  | *10% ad-valorem* | *3,500d/ liter* | *60d/ gr/100ml* | *7,000d/ liter* | *40% ad-valorem* |
| Weight change (kg) | | | | | | |
| *Children aged 2-17* | Boys | 0.06 | 0.11 | 0.23 | 0.22 | 0.23 |
|  | Girls | 0.06 | 0.12 | 0.24 | 0.23 | 0.24 |
| *Adults aged 18+* | Male | 0.10 (0.01) | 0.20 (0.01) | 0.41 (0.02) | 0.40 (0.02) | 0.42 (0.02) |
|  | Female | 0.13 (0.01) | 0.26 (0.01) | 0.53 (0.03) | 0.51 (0.02) | 0.53 (0.03) |
| Projected overweight prevalence (23 ≤ BMI <25) – Mean (SE) | | | | | | |
| Both sexes | | 17.7% (0.88%) | 17.4% (0.87%) | 13.4% (0.77%) | 13.2% (0.76%) | 13.3% (0.76%) |
| Male | | 19.5% (1.37%) | 19.0% (1.38%) | 15.1% (1.25%) | 14.9% (1.25%) | 15.0% (1.25%) |
| Female | | 15.9% (0.98%) | 15.9% (0.97%) | 11.7% (0.86%) | 11.6% (0.84%) | 11.7% (0.86%) |
| Projected obesity grade I prevalence (25 ≤ BMI <30) – Mean (SE) | | | | | | |
| Both sexes | | 13.3% (0.82%) | 13.1% (0.82%) | 9.9% (0.70%) | 10.4% (0.74%) | 10.0% (0.71%) |
| Male | | 12.6% (1.19%) | 12.6% (1.19%) | 10.4% (1.11%) | 10.8% (1.16%) | 10.4% (1.11%) |
| Female | | 13.9% (1.03%) | 13.5% (1.02%) | 9.5% (0.88%) | 10.0% (0.90%) | 9.6% (0.89%) |
| Projected obesity grade II prevalence (BMI ≥30) – Mean (SE) | | | | | | |
| Both sexes | | 3.4% (0.44%) | 3.4% (0.44%) | 3.0% (0.43%) | 3.0% (0.43%) | 3.0% (0.43%) |
| Male | | 1.6% (0.37%) | 1.6% (0.37%) | 1.3% (0.33%) | 1.3% (0.33%) | 1.3% (0.33%) |
| Female | | 5.1% (0.76%) | 5.1% (0.76%) | 4.7% (0.73%) | 4.7% (0.73%) | 4.7% (0.73%) |
| % reduction in diabetes prevalence | | | | | | |
| Both sexes | | 0.02% (0.000%) | 0.04% (0.000%) | 0.39% (0.003%) | 0.35% (0.002%) | 0.38% (0.003%) |
| Male | | 0.01% (0.000%) | 0.02% (0.000%) | 0.18% (0.004%) | 0.16% (0.003%) | 0.17% (0.004%) |
| Female | | 0.03% (0.000%) | 0.06% (0.000%) | 0.54% (0.004%) | 0.49% (0.004%) | 0.53% (0.004%) |
| Number of avoided diabetes case | | 14,299  (59) | 27,332  (118) | 252,582 (1,206) | 229,487 (1,089) | 247,404 (1,177) |
| Healthcare cost saving (mil. USD) | | 4.89 | 9.35 | 86.44 | 78.54 | 84.67 |
|  |  | (0.03) | (0.06) | (0.58) | (0.52) | (0.57) |
| Healthcare cost saving (bil. VND) | | 109.47 | 209.25 | 1,933.70 | 1,756.89 | 1,894.06 |
|  |  | (0.67) | (1.34) | (12.97) | (11.63) | (12.75) |
