# Supplementary material for: Estimating the health impacts of sugar-sweetened beverage tax for informing policy decisions about the obesity burden in Vietnam
Source: PLoS One. 2023 Apr 4;18(4):e0274928. doi: 10.1371/journal.pone.0274928 (PMC10072454; doi:10.1371/journal.pone.0274928)
Supplement: S3 File — (DOCX) [file pone.0274928.s003.docx]

# **Supplementary 3: Comparison of current findings with previous works**

**Table S4: Potential health impacts of SSB tax from previous simulation works**

| **Country** | **Model  characteristics** | **Tax scenario** | **Health benefits** |
| --- | --- | --- | --- |
| Current study | Micro, static; Adult 18-69; 1-3 years | Tax increase: 60đ/gr/l00ml  Price increase: 19%  PE = -1.14 | - Overweight *: ↓1.2%; - Obesity *: ↓ 0.20%; - Diabetes: ↓0.12% |
| Ireland (2013) (33) | Macro, static; Adults 18-75+; 3 years | Tax increase: 10% Price increase: 9% PE = -0.9 | - Obesity: ↓ 1.3% Overweight: ↓ 0.7% |
| India (2014) (34) | discrete micro simulation; hypothetical adult population aged 25-65; 10 years | Tax increase: 20% Price increase: 20% PE = -0.94 | - Overweight & obesity: ↓3.0% (1.6-5.9); - Diabetes: ↓1.6% (1.2-1.9) |
| South  African (2014) (18) | Macro, static; Adults aged 15+; 3 years | Tax increase: 20% Price increase: 20% PE = -1.299 | - Obesity prevalence: ↓3.8% (males); ↓2.4% (females) |
| Indonesia (2018) (19) | Macro, multistate life-table based Markov model; Population 0-95+ years old; Lifetime | Tax increase: approx. 20% Price increase: approx. 20% PE = -1.13 to -1.34 | - Overweight: ↓2.9% (males); ↓1.4% (females) - Obesity: ↓7.3% (males), ↓3.9% (females); |
| Zambia (2020)  (35) | Macro life-table based Markov model; Adults aged 15-65+; 40 years | Tax increase: 25% Price increase: 25% PE = -1.30 | - Obesity prevalence: ↓0.49% (0.41-0.57) |
| Thailand (2021) (16) | Macro, static; Children 3-17 & adults 18+; 1-3 years | Tax increase: 11%, 20%, 25% Price increase: 11%, 20%, 25% PE = 1.30 | - Obesity prevalence: ↓1.73%, ↓3.83%, and ↓4.91%, respectively |
| ** To be consistent with other studies, the study results on overweight is reported as of 25 ≤BMI<30 and obesity as of BMI≥30.* ↓ indicates the reduction on prevalence. | | | |
